# Grain iron and zinc density in pearl millet: combining ability, heterosis and association with grain yield and grain size

**DOI:** 10.1186/2193-1801-3-763

**Published:** 2014-12-26

**Authors:** Anand Kanatti, Kedar N Rai, Kommineni Radhika, Mahalingam Govindaraj, Kanwar L Sahrawat, Aluri S Rao

**Affiliations:** International Crops Research Institute for Semi-Arid Tropics (ICRISAT), Patancheru, Hyderabad, 502324 Telangana India; Department of Genetics and Plant Breeding, College of Agriculture, Professor Jayashankar Telangana State Agricultural University, Rajendranagar, Hyderabad, 500 030 Telangana India

**Keywords:** Pearl millet, Micronutrients, Grain yield, Genetic variability, Hybrids, Heterosis, Correlation

## Abstract

Genetics of micronutrients and their relationships with grain yield and other traits have a direct bearing on devising effective strategies for breeding biofortified crop cultivars. A line × tester study of 196 hybrids and their 28 parental lines of pearl millet (*Pennisetum glaucum* (L.) R.Br.) showed large genetic variability for Fe and Zn densities with predominantly additive gene action and no better-parent heterosis. Hybrids with high levels of Fe and Zn densities, involved both parental lines having significant positive general combining ability (GCA), and there were highly significant and high positive correlations between performance *per se* of parental lines and their GCAs. There was highly significant and high positive correlation between the Fe and Zn densities, both for performance *per se* and GCA. Fe and Zn densities had highly significant and negative, albeit weak, correlations with grain yield and highly significant and moderate positive correlation with grain weight in hybrids. These correlations, however, were non-significant in the parental lines. Thus, to breed hybrids with high Fe and Zn densities would require incorporating these micronutrients in both parental lines. Also, simultaneous selection for Fe and Zn densities based on performance *per se* would be highly effective in selecting for GCA. Breeding for high Fe and Zn densities with large grain size will be highly effective. However, combining high levels of these micronutrients with high grain yield would require growing larger breeding populations and progenies than breeding for grain yield alone, to make effective selection for desirable recombinants.

**Electronic supplementary material:**

The online version of this article (doi:10.1186/2193-1801-3-763) contains supplementary material, which is available to authorized users.

## Introduction

Micronutrient malnutrition resulting from dietary deficiency of one or more micronutrients has been recognized as a serious human health problem worldwide. The most striking of these are iron (Fe) and zinc (Zn) deficiencies that rank 9^th^ and 11^th^, respectively, among the top 20 risk factors contributing to global burden of disease (WHO^2002^). Pharmaceutical supplementation, industrial fortification and dietary diversification are some of the interventions that have been used to address this problem. Notwithstanding the recurring cost, the impact of supplementation and fortification in the developing countries remains limited because of poor infrastructure and delivery system (Stein et al.^2005^). Dietary diversification raises an issue of diverse food affordability since a sharp increase in food prices will have a large impact on poor households. It also has problem of consumer acceptance in case dietary diversification calls for including foods which are not a part of conventional diets. Biofortification of staple crops, especially for mineral micronutrients, is a sustainable and cost-effective approach. It has great promise for improving the mineral nutritional status and health of poor populations in both rural and urban areas of the developing world (Bouis^2003^). Biofortified cultivars of staple crops improved for mineral micronutrients are also readily acceptable to consumers as their adoption does not call for change in dietary habits.

Pearl millet (*Pennisetum glaucum (* L.) R. Br.) is a major warm-season cereal grown on 28 million ha for grain and fodder production in some of the most marginal areas of the arid and semi-arid tropical regions of Asia and Africa. In these regions, pearl millet is a major source of dietary energy and mineral micronutrients. India is the largest producer of this crop with >9 million ha area and 8.5 million tons of grain production (Yadav et al.^2012^). The contribution of pearl millet to the total Fe and Zn intake from all food sources has been reported to very widely vary across rural India. For instance, it was observed to be contributing 19-63% of the total Fe intake and 16-56% of the total Zn intake in parts of Rajasthan, Maharashtra and Gujarat states (Parthasarathy Rao et al.^2006^). Large genetic variability for Fe and Zn density observed in the breeding lines, improved populations and germplasm (Velu et al.^2007^,2008a; Rai et al.^2012^) provides for good prospects to breed improved pearl millet cultivars with elevated levels of these micronutrients. The International Crops Research Institute for the Semi-Arid Tropics (ICRISAT), supported by HarvestPlus Challenge Program of the Consultative Group on International Agricultural Research (CGIAR), and in partnership with the public and private sector breeding program in India, has undertaken a major initiative to develop high-yielding hybrids with high levels of Fe and Zn density in pearl millet.

Pearl millet is a highly cross-pollinated crop with open-pollinated varieties (OPVs) and hybrids as the two broad cultivar options. Hybrids are the most dominant cultivars in India, occupying >70% of area under improved pearl millet cultivars, with OPVs cultivated on limited scales. A preliminary study showed about two-fold differences for Fe and Zn densities among pearl millet hybrids under cultivation in India, with Fe density varying from 31 to 61 mg kg^-1^ and Zn density varying from 32 to 54 mg kg^-1^ (Rai et al.^2013^). An understanding of the nature of genetic variability and heterosis will have a direct bearing on devising effective hybrid breeding strategies for Fe and Zn density. There is limited information on genetic variability and heterosis for Fe and Zn density in pearl millet (Velu et al.2011b; Govindaraj et al.^2013^). While improving the Fe and Zn densities, it is important that genetic gains for these micronutrients are not made at the expense of grain yield and grain size. The main objective of this research was to examine the nature of genetic variability in relation to heterosis for Fe and Zn density. Since, there exists a wealth of literature on genetic variability and heterosis for grain yield and grain size (Khairwal et al.^1999^), genetic variability and heterosis for these two traits were studied in the specific context of their associations with Fe and Zn densities.

## Material and methods

### Genetic materials

Fourteen maintainer lines (B-lines) used to as female parents (F), 14 restorer lines (R-lines) used as male parents (M), and 196 hybrids produced by F × M crosses during the 2010 summer season were included in this study. The parental lines of the hybrids were of diverse parentage (Table 1) with wide range of Fe and Zn densities, and differed for grain yield and various agronomic traits such as plant height, tillering, panicle size and 1000-grain weight.

**Table 1:** **Parentage of inbred lines used in line (female) × tester (male) study**

ID^a^	Female/Male parents^b^	Parentage^c^
**Female parents**		
1	ICMB 88004	Togo-11-5-2 selection
2	ICMB 92111	(81B× 843B)-11-1-1-B
3	ICMB 92888	(843B× ICMPS 900-9-3-2-2)-41-2-6-2-2
4	ICMB 93222	(26B× 834B)-11-2-B-B
5	ICMB 97111	HTBC-48-B-1-1-1-1
6	ICMB 98222	ARD-288-1-10-1-2(RM)-5
7	ICMB 02555	ICMV 87901-175-2-3-2-B-1
8	ICMB 04888	[(843B× ICTP 8202-161-5)-20-3-B-B-3× B-lines bulk]-2-B-1-3
9	ICMB 05555	[(BSECBPT/91-39× SPF3/S91-116)-15-2-1-4-4× B-lines bulk]-1-B-4-1
10	ICMB 07555	[(843B× ICTP 8202-161-5)-20-3-B-B-3× B-lines bulk]-2-B-1
11	ICMB 07777	{ICMB 99555× [(78-7088/3/SER3 AD//B282/(3/4 EB) × PBLN/S95-359)-19-5-B-B]}-13-2-B-B-B-B
12	ICMB 07999	(HTBC-48-B-1-1-1-5× B-line bulk)-25-1-B-B
13	ICMB 08222	[78-7088/3/SER3 AD//B282/(3/4)EB× PBLN/S95-359]-19-2-B-1-B-B-3
14	ICMB 08333	[ICMB 97444× (843B× 405B)-4]-1-2-B-B-B
**Male parents**		
15	PRP 1	(EERC-HS-29)-B-12-4-1-1
16	PRP 2	(EERC-HS-34)-B-7-2-3-2
17	PRP 3	LaGrap C2-S1-38-2-1-1-1
18	PRP 4	(MC 94 C2-S1-3-2-2-2-1-3-B-B× SDMV 90031 S1-93-3-1-1-3-2-B-2)-B-23-2-2
19	PRP 5	AIMP 92901 S1-15-1-2-3-B-2-B-25-1-1
20	PRP 6	(MC 94 C2-S1-3-2-2-2-1-3-B-B× AIMP 92901 S1-488-2-1-1-4-B-B)-B-8-3-1-3-B-B
21	PRP 7	Jakhrana × SRC II S2-215-3-2-1-B-3
22	PRP 8	(ICMS 7704-S1-127-5-1 × RCB-2 Tall )-B-19-3-2-1-1-1-B
23	PRP 9	MRC S1-9-2-2-B-B-4-B-B
24	IPC 616	[J 260-1× 700557-1-4-10-5-1]-1-2-1-3
25	IPC 843	[(J 834× 700516)]-1-4-4-2-4
26	IPC 1178	(A 836× J 1798-32-2-2)-5-1-1
27	IPC 1354	EICP 8103–5 (Duplicate 001349)
28	IPC 390	(F4FC 1498-1-1-3× J 104)-11-2-1-1

^a^*ID* 1–14 female lines (B lines) and 15–28 male lines (R lines); ^b^*ICMB* ICRISAT Millet B-line; *PRP* Potential Restorer Parent; *IPC* ICRISAT Pollinator Collection; ^c^*ICMPS* ICRISAT Millet Pollinator for Smut resistance; *HTBC* High-Tillering B-Composite; *ARD* Appa Rao, Rai and Djaney; *ICMV* ICRISAT Millet Variety; *ICTP* ICRISAT Togo Patancheru; *BSECBPT* Bold-Seeded Early Composite Best Population Progeny Trial; *EB* Ex-Bornu; *SPF*_*3*_ Seed Parent F_3_ Progeny; *PBLN* Potential B-line Nursery; *EERC* Extra-Early Restorer Composite; *LaGrap* Large Grain Population; *MC* Medium Composite; SDMV SADCC Millet Variety; *AIMP* Aurangabad ICRISAT Millet Population; *ICMS* ICRISAT Millet Synthetic; *MRC* Mandor Restorer Composite; *EICP* Elite ICRISAT Pollinator.

### Field trials

The hybrids and parents were planted on 17-July during the 2011 rainy season (July to October) and on 24-January during 2012 summer season (January to April) at ICRISAT, Patancheru. Monthly mean temperatures varied from 20°C to 31°C and relative humidity from 50% to 93% during the rainy crop season. In the summer crop season, monthly mean temperatures varied from 16°C to 40°C and relative humidity from 22% to 79%. Both trials were planted in randomized complete block design (RCBD) with three replications but in adjacent blocks. Sowing was done by tractor-mounted 4-cone planter (7100 US model). Each entry was planted in two rows of 2 m length, spaced at 75 cm between rows in rainy season and 60 cm in summer season. Overplanted plots were thinned 15 d after planting to single plants spaced 15 cm apart within each row. A basal dose of 100 kg of diammonium phosphate (18% N and 46% P) was applied at the time of field preparation and 100 kg of urea (46% N) was applied as top dressing within 2 to 4 d after thinning. Fields were irrigated at 7 to 10 d intervals in summer crop season and twice in rainy crop season to ensure no moisture stress. All the recommended agronomic practices were followed for good crop growth. At the time of planting, four well spread representative soil samples were collected from the experimental fields from 0–30 cm layer and later bulked to prepare one composite sample for micronutrient analysis.

The plots of all the entries were harvested at or after physiological maturity (85–90 days after planting). During harvest, main panicles of five random plants from each plot were harvested and stored separately in a cloth bag to produce clean grain samples for micronutrient analysis. The remaining panicles of the plot were harvested as a bulk. These panicles were sundried for 10 to 15 days. While threshing, five separately harvested panicles mentioned above were manually threshed first and approximately 20 g of grains were collected for Fe and Zn analysis. The left over grains from these panicles were added to the bulk grain produced by threshing all the panicles of the plot in a multi-head machine thresher. The grain yield, including the 20 g sample taken for micronutrient analysis, was recorded for each plot and converted to t ha^-1^for grain yield analysis. A random sample of 200 grains for each entry was weighed to determine 1000-grain weight.

### Micronutrient analysis

Grain Fe and Zn densities were analyzed at the Charles Renard Analytical Laboratory, ICRISAT, Patancheru, India following the method described by Wheal et al. (2011). The ground samples were digested in closed tubes; and Fe and Zn in the digests were analyzed using Inductively Coupled Plasma Optical Emission Spectrometry (ICP-OES). Briefly, grain samples were finely ground and oven dried at 60°C for 48 h before analyzing them for Fe and Zn. Ground sample (0.2 g) was transferred to 25 mL polyprophelene PPT tubes; digestion was initiated by adding 2.0 mL of concentrated nitric acid (HNO_3_) and 0.5 mL of 30% hydrogen peroxide (H_2_0_2_). Tubes were vortexed to ensure the entire sample was wetted, and then pre-digested overnight at room temperature. Tubes were vortexed again before placing them into the digestion block and initially heated at 80°C for 1 hour, followed by digesting at 120°C for 2 hours. After digestion, the volume of the digest was made to 25 mL using distilled water; and the content was agitated for 1 minute by vortex mixer. The digests were filtered and Fe and Zn densities were determined using ICP-OES. Care was taken at each step to avoid any contamination of the grains with dust particles and any other extraneous matter (Stangoulis and Sison^2008^). The soil samples were analyzed for extractable Fe and Zn content by Atomic Absorption Spectroscopy (AAS) as described by Lindsay and Norvell (1978). The mean soil Fe and Zn contents extractable with Diethylene Triamine Pentaacetic Acid (DTPA) were respectively, 13.0 and 7.2 mg kg^-1^ in the rainy season, and 12.1 and 4.5 mg kg^-1^in the summer season. These Fe and Zn contents in the soil were far above the critical levels required by plants (2.6 to 4.5 mg kg^-1^ for Fe content, and 0.6 to 1.0 mg kg^-1^ for Zn content) (Tisdale et al.^1993^; Sahrawat and Wani^2013^).

### Statistical analysis

All the data were analyzed using Statistical Analysis Systems (SAS) version 9.2 (SAS Institute Inc.^2004^). ANOVA for individual environments and pooled ANOVA over the two environments were performed using Generalized Linear Model procedures following a random-effects model (Steel and Torrie^1980^; Hallauer and Miranda^1981^; McIntosh^1983^). All effects were considered as random in the combined analysis of variance and Satterthwaite’s approximation was used to obtain the appropriate degrees of freedom for the synthesized *F*-test (Satterthwaite^1941^,^1946^). Line × tester model for female × male hybrid (Kempthorne^1957^) was used to obtain estimates of general combining ability (GCA) for male and female parents as well as specific combining ability (SCA) effects for hybrids. From the analysis of combining ability ANOVA, variances attributed to general combining ability (σ^2^_GCA_) and specific combining ability (σ^2^_SCA_) were derived (Kaushik et al.^1984^) to estimate the predictability ratio 2σ^2^_GCA_/(2σ^2^_GCA_ + σ^2^_SCA_) following Baker (1978). The estimates of mid-parent heterosis (MPH) and better-parent heterosis (BPH) were derived for individual environments as well as for the mean of two environments following Hallauer and Miranda (1981). The tests of significance for MPH and BPH were done via “t” test. The Pearson correlation coefficient among the traits was calculated using the PROC CORR procedure.

## Results

### Parental line performance *per se*

There were highly significant differences among the parental lines (P) and their interaction with the environments (P × E) for Fe and Zn densities and 1000-grain weight (Table 2). For grain yield, only the differences among male parents (M) and interaction of female parents (F) with environment (F × E) were significant. However, the contribution of parent × environment interaction to the variability relative to those due to genetic differences among the parental lines was much smaller for Fe (14%), Zn (18%) and 1000-grain weight (17%), and was much higher for grain yield (66% ). Based on the mean performance over the two environments, large differences were observed among the parental lines for Fe and Zn densities. The Fe density varied from 30.3 to 77.2 mg kg^-1^ among the females and 32.0 to 82.1 mg kg^-1^ among the males, while Zn density varied from 27.4 to 45.3 mg kg^-1^ among the females and 29.0 to 55.5 mg kg^-1^ among the males (Table 3). The 1000-grain weight varied from 7.4 to 12.9 g among the females and from 6.9 to 11.5 g among the males, while grain yield varied from 1.6 to 3.6 t ha^-1^ among the females and 1.4 to 3.0 t ha^-1^ among the males.

**Table 2:** **Mean square for grain iron (Fe) and zinc (Zn) density, 1000-grain weight (GW) and grain yield (GY) of parental lines**

Source of variation	df	Mean square			
		Fe (mg kg^-1^)	Zn (mg kg^-1^)	GW(g)	GY (t ha^-1^)
Environment (E)	1	4017.4^**^	6002.5^**^	16.45^*^	28.07^**^
Replication /E	4	243.5^**^	11.2	0.17	0.58^**^
Parents (P)	27	958.1^**^	324.7^**^	18.36^**^	1.56
Female (F)	13	988.1^**^	222.3^**^	13.78^*^	1.74
Male (M)	13	1001.0^**^	410.1^**^	14.98^**^	1.14^**^
F vs M	1	11.7^**^	545.9^**^	121.68^**^	4.48^*^
P × E	27	137.4^**^	56.8^**^	3.20^**^	1.03^**^
F × E	13	148.7^**^	29.9^*^	4.20^**^	1.89^**^
M × E	13	136.1^**^	74.5^**^	1.33^**^	0.21
F vs M × E	1	7.5^**^	177.2^**^	14.44^**^	0.37
Error	108	25.6	13.4	0.41	0.14

^*,**^Significant at the P < 0.05 and 0.01 probability level, respectively.

**Table 3:** **Performance** ***per se*** **of parental lines and their general combining ability (GCA) effects**

ID^a^	Performance ***per se***^b^				GCA effects			
	Fe (mg kg^-1^)	Zn (mg kg^-1^)	GW (g)	GY (t ha^-1^)	Fe (mg kg^-1^)	Zn (mg kg^-1^)	GW (g)	GY (t ha^-1^)
**Female parents**								
1	52.6	40.0	11.0	2.9	4.1^**^	2.5^**^	0.6^**^	-0.03^NS^
2	30.3	27.4	7.4	2.2	-9.5^**^	-4.2^**^	-1.5^**^	0.12^*^
3	39.1	33.2	12.8	2.3	-4.8^**^	-2.9^**^	0.4^**^	0.04^NS^
4	58.6	40.9	11.4	3.1	4.8^**^	3.6^**^	0.5^**^	0.33^**^
5	39.1	30.6	12.9	3.6	-5.1^**^	-4.0^**^	0.1^NS^	0.01^NS^
6	77.2	45.3	10.2	2.6	7.3^**^	2.4^**^	-0.3^**^	-0.08^NS^
7	43.9	37.1	10.3	3.2	0.8^NS^	0.1^NS^	-0.7^**^	0.05^NS^
8	55.3	45.0	9.2	1.6	1.8^**^	1.6^**^	0.7^**^	-0.23^**^
9	63.2	44.0	11.2	2.0	7.6^**^	3.7^**^	1.0^**^	0.01^NS^
10	51.1	40.0	10.9	2.3	-1.8^**^	0.5^NS^	0.9^**^	0.11^**^
11	43.7	32.7	8.5	2.4	-3.4^**^	-3.7^**^	-0.6^**^	-0.05^NS^
12	32.1	27.6	11.0	2.8	-6.6^**^	-2.6^**^	-0.4^**^	0.29^**^
13	59.8	40.7	10.8	2.2	6.4^**^	2.5^**^	0.5^**^	-0.37^**^
14	48.5	38.2	9.6	2.3	-1.6^**^	0.6^NS^	-1.0^**^	-0.26^**^
**Male parents**								
15	43.2	41.7	9.4	2.6	-1.4^**^	2.1^**^	0.7^**^	0.22^**^
16	82.1	55.5	8.8	1.8	6.4^**^	3.1^**^	-0.3^**^	-0.20^**^
17	63.9	49.5	11.5	2.2	9.5^**^	6.2^**^	0.9^**^	-0.44^**^
18	52.1	41.5	11.2	3.0	3.9^**^	0.5^NS^	0.7^**^	-0.01^NS^
19	57.1	46.0	8.5	2.4	5.6^**^	3.8^**^	0.1^NS^	0.04^NS^
20	41.6	33.3	11.0	2.4	-2.5^**^	-2.9^**^	0.6^**^	0.24^**^
21	40.0	35.6	10.0	2.6	-2.2^**^	-1.2^**^	0.2^**^	0.10^*^
22	46.1	37.8	7.6	2.3	1.7^**^	1.8^**^	0.3^**^	-0.07^NS^
23	43.1	31.5	6.9	2.0	-9.2^**^	-7.8^**^	-1.6^**^	0.47^**^
24	56.3	44.8	7.0	1.8	1.8^**^	0.2^NS^	-0.8^**^	0.05^NS^
25	57.8	49.6	8.3	2.7	1.1^*^	2.8^**^	-0.2^**^	-0.11^NS^
26	44.4	44.4	7.5	1.7	-0.5^NS^	1.8^**^	-0.1^NS^	0.14^**^
27	32.0	29.0	8.0	1.4	-7.5^**^	-5.0^**^	0.4^**^	-0.30^**^
28	39.0	29.4	7.2	2.1	-6.6^**^	-5.1^**^	-0.9^**^	-0.14^*^
SE ± ^c^	2.06	1.49	0.26	0.15	0.59	0.42	0.07	0.05
LSD^d^	5.79	4.19	0.73	0.43				

^a^Refers to Table 1; ^b^Mean of two environments (2011 rainy season and 2012 summer season) for grain iron (Fe) and zinc (Zn) density, 1000-grain weight (GW) and grain yield (GY); ^*c*^*SE* Standard error; ^d^*LSD* Least significant difference; ^*, **^Significant at the 0.05 and 0.01 probability level, respectively; ^*NS*^Non-significant.

### Genetic variability

**Figure 1:**
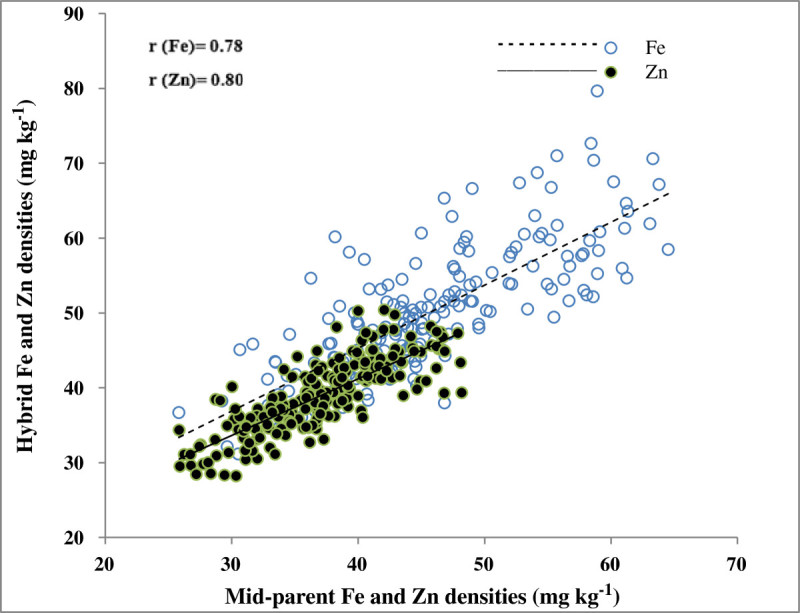
**Relationship between mid-parental and hybrid values.** Mean of two environment for grain iron (Fe) and zinc (Zn) densities.

The differences among hybrids (H) as well their interaction with environment (H × E) were highly significant for all four traits (Table 4). However, the contribution of H × E interaction to the variability relative to those due to genetic differences among the hybrids were only 14-16% for Fe and Zn density and 1000- grain weight and much higher (41%) for grain yield. Further partitioning of H × E interaction showed that contribution of F × E interaction to the variability relative to that due to F effect was 7-11% for Fe and Zn density and 1000-grain weight, while it was much higher (29%) for grain yield. The contribution of M × E interaction to the variability relative to that due to M effect was 4-6% for both micronutrients and 1000-grain weight, while it was 24% for grain yield. Clearly, the variability attributable to interaction of F and M with the environments was much smaller as compared to those attributable to F and M effects, respectively, for all the traits except for grain yield. The contribution of F × M × E interaction to variability relative to that due to F × M interaction was 40-59% for both micronutrients, 1000-grain weight and grain yield, which were all highly significant. However, the contribution of F × M interaction effect to variability relative to those due to F and M effects combined was only about 20% for Fe and Zn density and 26% for 1000-grain weight, while it was about 20% higher for grain yield. As a result, the variance due to general combining ability (σ^2^_GCA_) was about 4 to 5 times higher than the variance due to specific combining ability (σ^2^_SCA_) for Fe and Zn density, and more than twice for 1000-grain weight. For grain yield, the σ^2^_GCA_ was 50% of the σ^2^_SCA_. This led to predictability ratio close to unity for both micronutrients (0.90 for Fe and 0.91 for Zn). Compared to these micronutrients, the predictability ratio was slightly lower for 1000-grain weight (0.84) and much lower for grain yield (0.52). There were highly significant and high positive correlations between the mid-parental values and hybrid performance *per se* for Fe density (r = 0.78) and Zn density (r = 0.80) (Figure 1). Though highly significant and positive, these correlations were moderate for 1000-grain weight (r = 0.61) and low for grain yield (r = 0.23).

**Table 4:** **Mean square for hybrids and genetic components**

Source of variation	df	Mean square^a^			
		Fe (mg kg^-1^)	Zn (mg kg^-1^)	GW(g)	GY (t ha^-1^)
Environment (E)	1	22524.5^**^	48885.5^**^	313.2^**^	25.12^**^
Replication/E	4	62.6	858.3^**^	9.1^**^	19.26^**^
Hybrids (H)	195	401.7^**^	164.7^**^	7.7^**^	1.15^**^
Female (F)	13	2574.9^**^	720.9^**^	48.6^**^	3.27^*^
Male (M)	13	2470.6^**^	1325^**^	42.9^**^	4.66^**^
F × M	169	77.0^**^	32.9^**^	1.8^**^	0.72^**^
H × E	195	55.4^**^	26.2^**^	1.1^**^	0.47^**^
F × E	13	174.8^**^	62.3^**^	5.3^**^	0.93^**^
M × E	13	111.9^**^	72.9^**^	2.1^**^	1.12^**^
F × M × E	169	41.6^**^	19.7^*^	0.7^**^	0.38^**^
Error	780	29.5	15.1	0.4	0.25
**Genetic components**					
σ ^2^_GCA_^b^		27.9	11.2	0.49	0.03
σ ^2^_SCA_^c^		5.9	2.2	0.18	0.06
PR = (2σ ^2^_GCA_)/(2σ ^2^_GCA_ + σ^2^_SCA_)		0.90	0.91	0.84	0.52

^a^Grain iron (Fe) and zinc (Zn) density, 1000-grain weight (GW) and grain yield (GY); ^b^*σ*^*2*^_*GCA*_ Variance attributed to general combining ability; ^c^*σ*^*2*^_*SCA*_ Variance attributed to specific combining ability; *PR* Predictability ratio; ^*,**^Significant at the 0.05 and 0.01 probability level, respectively.

### General combining ability (GCA) of parental line

Amongst the 14 female parents, 6 lines had significant positive GCA for Fe density and most of these had >55 mg kg^-1^ Fe density, while 7 females had significant negative GCA and most of these had <44 mg kg^-1^Fe density (Table 3). The two best general combiners were ICMB 98222 (ID 6) and ICMB 05555 (ID 9) that also had the highest Fe densities of 77.2 and 63.2 mg kg^-1^, respectively. Among the 14 male parents, 7 lines had significant positive GCA and most of these had >56 mg kg^-1^Fe density, while 6 lines had significant negative GCA and all these had <44 mg kg^-1^ Fe density. The two best general combiners were PRP 3 (ID 17) and PRP 2 (ID 16) that also had the highest Fe densities of 63.9 and 82.1 mg kg^-1^, respectively. Female and male lines considered together, there was highly significant and high positive correlation between the Fe density of parental lines *per se* and their GCA for this trait (*r* = 0.86; *p* <0.01). Broadly similar patterns were observed for the Zn density. Thus, 6 female parents had significant and positive GCA and all these females had ≥40 mg kg^-1^ Zn density, while 5 female parents had significant negative GCA and all these females had <34 mg kg^-1^ Zn density. Seven male parents had significant and positive GCA and most of these had >41 mg kg^-1^Zn density, while 5 male parents had significant negative GCA and all of these had <36 mg kg^-1^ Zn density. There was highly significant and high positive correlation between the Zn density of parental lines *per se* and their GCA for this trait as well (r = 0.85; *p* <0.01). Such a strong relationship between the performance *per se* of the parental lines and their GCA was not observed for 1000-grain weight (r = 0.59) and there was no significant correlation between these two performance parameters for grain yield.

### Specific combining ability (SCA) of hybrids

**Figure 2:**
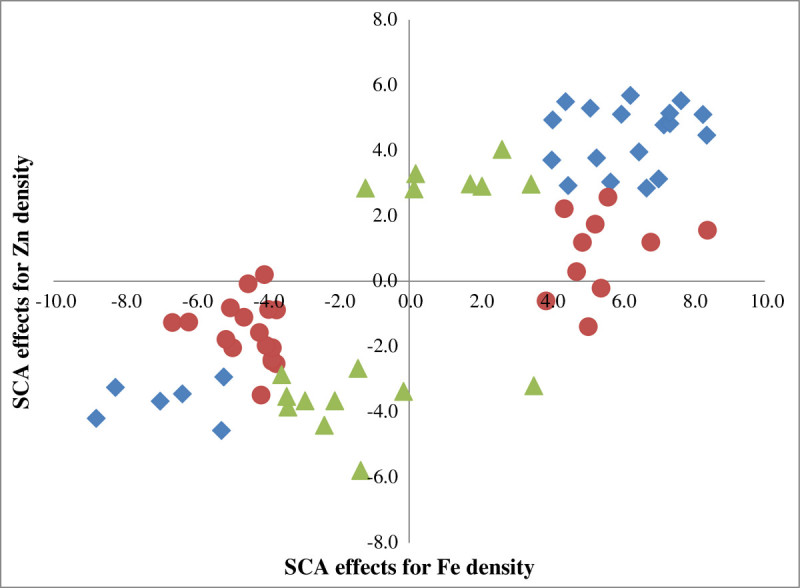
**Pearl millet hybrids with significant SCA effects.** Significant SCA for Fe density (red circle symbol), for Zn density (yellow triangle symbol) and both for Fe and Zn densities (blue diamond symbol).

Amongst 196 hybrids, 51 hybrids had significant SCA for Fe density, of which 28 were positive and 23 were negative (Figure 2). Similarly, 42 hybrids had significant SCA for Zn density, of which 25 were positive and 17 were negative. Of these, 18 hybrids had significant positive SCA effects, both for Fe and Zn densities. There were moderate but highly significant positive correlations between performance *per se* of hybrids and their SCA, both for Fe density (r = 0.41, *p* <0.01) and Zn density (r = 0.42, *p* <0.01). Seventy seven hybrids had significant SCA for 1000-grain weight, of which 37 were positive and 40 were negative. There was moderate but highly significant and positive correlation between 1000-grain weight of the hybrids *per se* and their SCA (r = 0.45, *p* <0.01). Fifty six hybrids had significant SCA for grain yield, of which 28 were positive and other 28 were negative, with highly significant and high positive correlation between the performance *per se* of the hybrids and their SCA (r = 0.74, *p* <0.01).

### Heterosis and hybrid performance

Averaged over the environments, Fe density among the hybrids varied from 25.8 to 64.5 mg kg^-1^, but none of the hybrids showed significant better-parent heterosis. However, 62 hybrids had significant mid-parent heterosis, of which 3 were positive and 59 were negative. Top 5% of the high-Fe hybrids had 60.2-64.5 mg kg^-1^ Fe density, which were not significantly different from each other, while the parental lines of these hybrids had large differences, varying from 46.1 to 77.2 mg kg^-1^ Fe density (Table 5). The perusal of parental combinations of these 10 high-Fe hybrids showed that both parents in all these hybrids had significant positive GCA, with at least one of the parents of all 10 hybrids having high Fe density (≥58.6 mg kg^-1^). In contrast, all the 5% bottom-Fe hybrids had ≤32.8 mg kg^-1^ Fe density, which, among themselves, were not significantly different from each other. However, all of these 10 hybrids were significantly different from the top 10 high-Fe hybrids. Both parents of the nine low-Fe hybrids and one of the parents in one hybrid had significant negative GCA, and most of the parents had <45 mg kg^-1^ Fe density.

**Table 5:** **Performance** ***per se*** **of hybrids and parental lines, and GCA of parents for 5% top and 5% bottom ranking hybrids**

Fe (mg kg^-1^)								Zn (mg kg^-1^)							
Hybrid^a^			Performance ***per se***^b^			GCA^c^		Hybrid^a^			Performance ***per se***^b^			GCA^c^	
P_1_		P_2_	F_1_	P_1_	P_2_	P_1_	P_2_	P_1_		P_2_	F_1_	P_1_	P_2_	P_1_	P_2_
**Top 5% hybrids**															
13	×	19	64.5	59.8	57.1	6.4^**^	5.6^**^	4	×	22	48.2	40.9	37.8	3.6^**^	1.8^**^
6	×	19	63.8	77.2	57.1	7.3^**^	5.6^**^	13	×	19	48.1	40.7	46.0	2.5^**^	3.8^**^
6	×	17	63.3	77.2	63.9	7.3^**^	9.5^**^	8	×	17	47.9	45.0	49.5	1.6^**^	6.2^**^
13	×	17	63.1	59.8	63.9	6.4^**^	9.5^**^	1	×	25	46.9	40.0	49.6	2.5^**^	2.8^**^
9	×	17	61.3	63.2	63.9	7.6^**^	9.5^**^	13	×	22	46.8	40.7	37.8	2.5^**^	1.8^**^
9	×	22	61.2	63.2	46.1	7.6^**^	1.7^**^	9	×	17	46.7	44.0	49.5	3.7^**^	6.2^**^
6	×	18	61.2	77.2	52.1	7.3^**^	3.9^**^	13	×	17	46.5	40.7	49.5	2.5^**^	6.2^**^
4	×	17	61.1	58.6	63.9	4.8^**^	9.5^**^	6	×	17	46.5	45.3	49.5	2.4^**^	6.2^**^
13	×	18	60.9	59.8	52.1	6.4^**^	3.9^**^	4	×	17	46.4	40.9	49.5	3.6^**^	6.2^**^
6	×	25	60.2	77.2	57.8	7.3^**^	1.1^*^	6	×	25	46.2	45.3	49.6	2.4^**^	2.8^**^
**Bottom 5% hybrids**															
5	×	23	32.8	39.1	43.1	-5.1^**^	-9.2^**^	5	×	28	28.1	30.6	29.4	-4.0^**^	-5.1^**^
2	×	20	31.8	30.3	41.6	-9.5^**^	-2.5^**^	5	×	27	27.8	30.6	29.0	-4.0^**^	-5.0^**^
14	×	23	31.7	48.5	43.1	-1.6^**^	-9.2^**^	3	×	23	27.5	33.2	31.5	-2.9^**^	-7.8^**^
10	×	28	30.7	51.1	39.0	-1.8^**^	-6.6^**^	11	×	23	27.4	32.7	31.5	-3.7^**^	-7.8^**^
2	×	28	30.6	30.3	39.0	-9.5^**^	-6.6^**^	2	×	28	27.2	27.4	29.4	-4.2^**^	-5.1^**^
3	×	27	30.5	39.1	32.0	-4.8^**^	-7.5^**^	12	×	23	26.8	27.6	31.5	-2.6^**^	-7.8^**^
2	×	27	30.5	30.3	32.0	-9.5^**^	-7.5^**^	3	×	27	26.7	33.2	29.0	-2.9^**^	-5.0^**^
12	×	27	29.7	32.1	32.0	-6.6^**^	-7.5^**^	5	×	23	26.2	30.6	31.5	-4.0^**^	-7.8^**^
2	×	22	29.2	30.3	46.1	-9.5^**^	1.7^**^	2	×	23	25.9	27.4	31.5	-4.2^**^	-7.8^**^
2	×	23	25.8	30.3	43.1	-9.5^**^	-9.2^**^	7	×	23	25.8	37.1	31.5	0.1^NS^	-7.8^**^
SE ±^d^			2.22	2.06	2.06	0.59	0.59				1.59	1.49	1.49	0.42	0.42
LSD^e^			6.16	5.79	5.79						4.40	4.19	4.19		

^a^*1-28* ID of inbred lines detailed in the Table 1; ^b^Mean performance at two environments (2011 rainy season and 2012 summer season) for grain iron (Fe) and zinc (Zn) density. ^c^*GCA* General combining ability effects; ^d^*SE* Standard error; ^e^*LSD* Least significant difference; ^* , **^Significant at the 0.05 and 0.01 probability level, respectively; ^*NS*^Non-significant.

Patterns for Zn density were similar to those for the Fe density. The Zn density among the hybrids varied from 25.8 to 48.2 mg kg^-1^, but only two of these hybrids had significant better-parent heterosis. Forty five hybrids (23%) had significant mid-parent heterosis, of which 6 were positive and 39 were negative. The top 5% high-Zn hybrids had 46.2-48.2 mg kg^-1^ Zn density, which were not significantly different from each other, while the parental lines of these hybrids had large differences, varying from 37.8 to 49.6 mg kg^-1^ Zn density (Table 5). The perusal of parental combination of these hybrids showed both parents with significant positive GCA and at least one parents with high Zn density (≥40.7 mg kg^-1^). Conversely, 5% bottom-Zn hybrids had low range of Zn density (25.8-28.1 mg kg^-1^), which, among themselves, were not significantly different from each other. However, all of these were significantly different from the top 5% high-Zn hybrids. Both parents of the nine low-Zn hybrids and one parent in one hybrid had significant negative GCA. The 1000-grain weight of hybrids varied from 8.8 to 14.3 g with significant better- parent heterosis observed in 110 hybrids. Similarly, grain yield of hybrids varied from 2.3 to 5.0 t ha^-1^, with significant better-parent heterosis observed in 178 hybrids.

### Character association

There was a highly significant and high positive correlation (r = 0.88, *p* <0.01) between the Fe and Zn density in the parental lines as well as in the hybrids (Table 6). Neither Fe nor Zn densities were correlated with grain yield or with 1000-grain weight in the parental lines. However, both micronutrients had highly significant and moderate positive correlation with 1000-grain weight (r = 0.42 and 0.43, *p* <0.01) and significant but week negative correlation with grain yield (r = -0.29 and -0.26, *p* <0.01) in the hybrids. Highly significant and moderate positive correlation between 1000-grain weight and grain yield was observed in the parental lines (r = 0.58, *p* <0.01), but it was not significant in hybrids. Interestingly, highly significant and high positive correlation was observed between the GCA of Fe and GCA of Zn density in parental lines (r = 0.91, *p* <0.01), and SCA of Fe and SCA of Zn density in hybrids (r = 0.71, *p* <0.01). Moderate and highly significant positive correlation was observed between the GCA of 1000-grain weight and Fe density (r = 0.51, *p* <0.01) and between the GCA of 1000-grain weight and Zn density (r = 0.54, *p* <0.01). The GCA of grain yield had significant but moderate negative correlation with Fe density (r = -0.41, *p* <0.01), but it was uncorrelated with GCA of Zn density. The SCAs of both micronutrients were not correlated with the SCAs of either 1000-grain weight or grain yield, but there was highly significant and weak positive correlation between the SCA of 1000-grain weight and grain yield (r = 0.21, *p* <0.01).

**Table 6:** **Correlation coefficient among traits in hybrids (above diagonal) and parents (below diagonal)**

Trait^a^	Fe (mg kg^-1^)	Zn (mg kg^-1^)	GY (t ha^-1^)	GW(g)
Fe	1	0.88^**^ (0.71^**^)	-0.29^**^ (-0.13^NS^)	0.42^**^ (0.003^NS^)
Zn	0.88^**^ (0.91^**^)^b^	1	-0.26^**^ (-0.14^NS^)	0.43 ^**^(-0.02^NS^)
GY	-0.11^NS^ (-0.41^*^)	-0.18^NS^ (-0.35^NS^)	1	-0.05^NS^ (0.21^**^)
GW	0.11 ^NS^(0.51^**^)	0.002 ^NS^ (0.54^**^)	0.58^**^ (-0.21^NS^)	1

^a^Grain iron (Fe) and zinc (Zn) density, 1000-grain weight (GW) and grain yield (GY); ^b^Values outside the parentheses are phenotypic correlations for performance *per se* and values within the parentheses are correlations between GCA effects in parents and between SCA effects in hybrids; ^*,**^Significant at the 0.05 and 0.01 probability level, respectively; ^*NS*^Non-significant.

## Discussion

Parental lines (both female and male parents) of diverse parentage included in this study had highly significant and large differences for Fe and Zn densities, and 1000-grain weight, with only male parents having significant differences for grain yield. Hybrids produced from female × male crosses had, as expected, highly significant and large differences for all four traits. The variability among the hybrids attributable to general combining ability (σ^2^_GCA_) was 4–5 times greater than the variability attributable to specific combining ability (σ^2^_SCA_) for Fe and Zn densities, with the predictability ratio being closer to unity for both micronutrients. This showed that both Fe and Zn densities were predominantly under additive genetic control. Earlier studies in pearl millet (Velu et al.2011b; Govindaraj et al.^2013^), rice (Zhang et al.^2004^) and maize (Gorsline et al.^1964^; Arnold and Bauman^1976^; Brkic et al.^2003^; Long et al.^2004^; Chen et al.^2007^) have also reported these micronutrients largely under additive genetic control. Highly significant and high positive correlations between performance *per se* of the hybrids and mid-parental values provided further support for these micronutrients being largely under additive genetic control. Similar results in pearl millet have been reported in earlier studies (Velu et al.2011b; Govindaraj et al.^2013^). The predominance of additive gene action would make recurrent selection for intra-population improvement and open–pollinated variety (OPV) development highly effective. However, development of hybrids with high Fe and Zn density would require that the same genes for Fe and Zn density are incorporated into both parental lines of the hybrids, since hardly any hybrid was found transgressing the parental lines for higher Fe density (i.e., no better-parent heterosis). About 30% of the hybrids, had significant mid-parent heterosis for Fe density; and 23% hybrids had significant mid-parent heterosis for Zn density, which is not unexpected as significant, albeit low, variability among the hybrids attributable to σ^2^_SCA_ had been observed for both micronutrients. Most of these mid-parent heterosis values were in the negative direction, indicating the involvement of genes other than those with additive gene action where alleles determining lower Fe and Zn densities are partially dominant. It is also likely that effects of genes acting additively for Fe and Zn densities are influenced by genetic backgrounds, the more so in the negative direction, mimicking low levels of partial dominance. *Iniadi g* ermplasm so far has been found to be the best source of high Fe and Zn density in pearl millet (Velu et al.2011b; Rai et al.^2012^; Govindaraj et al.^2013^). Thus, taking into consideration the additive gene action, if the same source is used to introgress the genes responsible for Fe and Zn density in both parental lines, it is likely to reduce the genetic diversity between B-lines (and consequently A-lines) and R-lines for other traits. This would potentially lead to reduction in heterosis for grain yield and other traits of agronomic and economic importance, which are predominantly under non-additive gene effects. Genomics approaches for selective introgression of genes for Fe and Zn density in the parental lines without disrupting the diversity between them for other traits can play a major role in breeding high-yielding hybrids with higher levels of Fe and Zn densities.

Hybrids in the top 5% ranks for Fe density involved generally both and always at least one parent that had positive GCA for this trait. A similar pattern was observed for Zn density where at least one of the parents of the top ranking 5% hybrids was a positive general combiner. There were highly significant and high positive correlations between performance *per se* of the inbred lines and their GCA both for Fe density and Zn density, indicating that selection for performance *per se* would be highly effective in selecting for the GCA of these micronutrients. Interestingly, GCA of Fe density also had highly significant and high positive correlation with the GCA of Zn density, which was not unexpected considering that the performance *per se* of the parental lines and their GCAs were highly significantly and positively correlated, and there was highly significant and high positive correlation between the Fe and Zn densities in the parental lines as well as in the hybrids. Similar relationships between these micronutrients have been reported in earlier studies on pearl millet (Velu et al.2008a,2008b; Gupta et al.^2009^; Rai et al.^2012^; Govindaraj et al.^2013^), and in other cereals, such as sorghum (Ashok Kumar et al.^2010^,^2013^), maize (Arnold et al.^1977^; Oikeh et al.^2003^,^2004^), rice (Stangoulis et al.^2007^; Anandan et al.^2011^), wheat (Garvin et al.^2006^; Peleg et al.^2009^; Zhang et al.^2010^; Velu et al.2011a), and finger millet (Upadhyaya et al.^2011^). Genomic studies in wheat (Peleg et al.^2009^; Singh et al.^2010^), rice (Stangoulis et al.^2007^), common bean (Cichy et al.^2009^; Blair et al.^2009^) and pearl millet (Kumar^2011^) have identified common and overlapping quantitative trait loci (QTL) for Fe and Zn densities. Thus, the patterns for Fe and Zn densities were similar whether it is with respect to the nature of genetic variability and heterosis, or with respect to the relationship between the parental lines and their GCA. This could likely result from similar physiological processes involved at one or more stages from soil uptake to loading in the grains, and tight linkage of some of the genes contributing to the major part of genetic variability for these micronutrients. It would appear that effective simultaneous selection for Fe and Zn densities in pearl millet can be made with respect to all these performance parameters, and application of genomics tools can significantly accelerate this process.

High predictability ratio and highly significant and high positive correlation between the mid-parental values and hybrid performance was indicative of predominantly additive genetic variation for 1000-grain weight. In contrast, moderate predictability ratio and low, though significant, correlation between the mid-parental values and hybrid performance was indicative of predominantly non-additive genetic variation for grain yield. Several studies in pearl millet have reported predominantly additive genetic variability for 1000-grain weight and predominantly non-additive genetic variability for grain yield (Khairwal et al.^1999^). The relative importance of additive and non-additive variation for these two traits were also reflected in heterosis patterns as 56% of the hybrids had significant better-parent heterosis with highly significant but moderate correlation between the hybrid performance *per se* and SCA for 1000-grain weight. In comparison, 91% hybrids showed significant better-parent heterosis for grain yield and there was highly significant and high positive correlation between the performance of hybrids *per se* and their SCA.

Both Fe and Zn densities were not correlated either with 1000-grain weight or with grain yield in the parental lines, which indicates that parental lines with high Fe and Zn density can be developed without compromising on grain size and grain yield. In hybrids, however, Fe and Zn densities had a highly significant and moderate positive correlation with 1000-grain weight. While some of the earlier studies in pearl millet (Velu et al.^2007^,2008a,2008b) have reported significant positive correlation of Fe and Zn with 1000-grain weight, other studies have reported no correlation of Fe and Zn densities with 1000-grain weight ( Gupta et al.^2009^; Rai et al.^2012^). This indicated that high Fe and Zn densities can be easily combined with large grain size. The Fe and Zn densities had highly significant though weak negative correlation with grain yield. Earlier studies in pearl millet hybrids (Rai et al.^2012^), wheat (Garvin et al.^2006^; Morgounov et al.^2007^; Shi et al.^2008^; Zhao et al.^2009^), sorghum (Reddy et al.^2005^) and maize (Bänziger and Long^2000^) reported significant negative relationship between micronutrients and yield. In the present study, however, these correlations were weak enough in the magnitude, indicating that if these were the results of adverse genetic associations, high-yielding hybrids with high Fe and Zn densities can be bred by making selection for these traits in larger segregating populations and progenies as compared to those used for breeding for grain yield alone. These weak negative relationships resulting from dilution effects, however, cannot be ruled out and, therefore, this subject merits further investigation.

## Conclusions

Genetic variability both for Fe and Zn densities was predominantly under additive genetic control, implying that intra-population improvement for these micronutrients is likely to be highly effective. However, breeding hybrids with high levels of Fe and Zn densities will require incorporating them in both parental lines, and application of genomics tools may significantly accelerate this process. Highly significant and high positive correlation between performance *per se* of parental line and their general combining ability (GCA) both for Fe and Zn densities showed that parental lines of potential hybrids with high GCA can be effectively selected based on their performance *per se*, thus enhancing the breeding efficiency. Lack of correlation of Fe and Zn densities with grain yield in inbred lines, but significant negative (although low) correlation in hybrids merits further investigation as these results have direct bearing on the efficiency of breeding high-yielding hybrids with high levels of Fe and Zn densities.

## Authors’ details

AK: Ph.D. Research Scholar and KR (Ph.D.): Associate Professor at Professor Jayashankar Telangana State Agricultural University, Rajendranagar, Telangana, India; KNR (Ph.D.) and MG (Ph.D.): Millet Breeders; KLS (Ph.D.): Soil Scientist, ASR (M.Sc.): Scientific Officer at International Crops Research Institute for the Semi-Arid Tropics (ICRISAT), Telangana, India.

## Authors’ original submitted files for images

Below are the links to the authors’ original submitted files for images. Authors’ original file for figure 1 Authors’ original file for figure 2

## Abbreviations

Fe: Iron
Zn: Zinc
PR: Predictability ratio
GCA: General combining ability
SCA: Specific combining ability
r: Correlation coefficient.
