# Supplementary figures and images for: Grain iron and zinc density in pearl millet: combining ability, heterosis and association with grain yield and grain size
Source: Springerplus. 2014 Dec 26;3:763. doi: 10.1186/2193-1801-3-763 (PMC4320223; doi:10.1186/2193-1801-3-763)

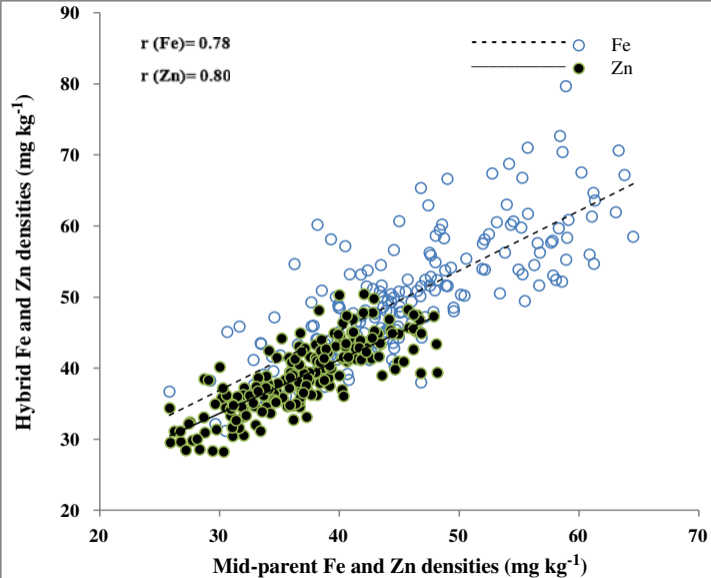

Supplement: Supplementary file 1 — Authors’ original file for figure 1 [file 40064_2014_1517_MOESM1_ESM.pdf]

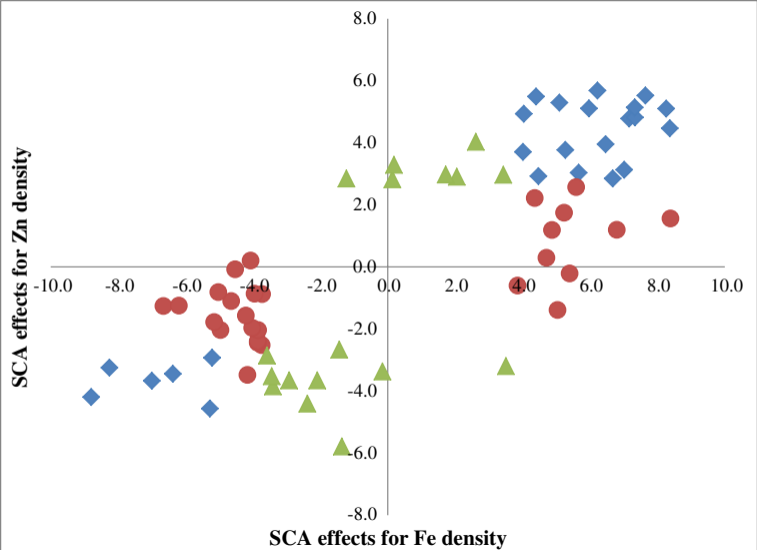

Supplement: Supplementary file 2 — Authors’ original file for figure 2 [file 40064_2014_1517_MOESM2_ESM.pdf]
